# Effect of Tau Fragment and Membrane Interactions on Membrane Permeabilization and Peptide Aggregation

**DOI:** 10.3390/membranes15070208

**Published:** 2025-07-13

**Authors:** Majedul Islam, Md Raza Ul Karim, Emily Argueta, Mohammed N. Selim, Ewa P. Wojcikiewicz, Deguo Du

**Affiliations:** 1 Department of Chemistry and Biochemistry, Florida Atlantic University, Boca Raton, FL 33431, USA; 2 Department of Biomedical Science, Charles E. Schmidt College of Medicine, Florida Atlantic University, Boca Raton, FL 33431, USA

**Keywords:** tau, lipid, membrane damage, protein aggregation, amyloid

## Abstract

Aggregation of tau protein is a hallmark feature of tauopathies such as Alzheimer’s disease. The microtubule-binding domain of tau plays a crucial role in the tau aggregation process. In this study, we investigated the dual effects of membrane interactions of tau_298–317_, a fragment peptide from the microtubule-binding domain, on peptide-induced membrane disruption and membrane-mediated peptide self-assembly. Our results show that neither wild-type tau_298–317_ nor its P301L or Ser305-phosphorylated mutants aggregate in the presence of zwitterionic POPC vesicles or cause lipid vesicle leakage, indicating weak peptide–membrane interactions. In contrast, tau_298–317_ strongly interacts with negatively charged POPG liposomes, leading to a rapid transition of the peptide conformation from random coils to α-helical intermediate conformation upon membrane adsorption, which may further promote peptide self-association to form oligomers and β-sheet-rich fibrillar structures. Tau_298–317_-induced rapid POPG membrane leakage indicates a synergistic process of the peptide self-assembly at the membrane interface and the aggregation-induced membrane disruption. Notably, phosphorylation at Ser305 disrupts favorable electrostatic interactions between the peptide and POPG membrane surface, thus preventing peptide aggregation and membrane leakage. In contrast, the P301L mutation significantly enhances membrane-mediated peptide aggregation and peptide-induced membrane disruption, likely due to alleviation of local conformational constraints and enhancement of local hydrophobicity, which facilitates fast conformational conversion to β-sheet structures. These findings provide mechanistic insights into the molecular mechanisms underlying membrane-mediated aggregation of crucial regions of tau and peptide-induced membrane damage, indicating potential strategies to prevent tau aggregation and membrane rupture by targeting critical electrostatic interactions between membranes and key local regions of tau.

## 1. Introduction

Tau is a microtubule-associated protein predominantly expressed in neurons. It plays a critical role in promoting microtubule assembly and maintaining cytoskeletal integrity [1,2]. Under pathological conditions, however, tau is subject to abnormal post-translational modifications such as hyperphosphorylation, leading to its dissociation from microtubules and subsequent aggregation into insoluble β-sheet-rich amyloid fibrils [3,4,5,6]. Neurofibrillary tangle deposition of tau represents a histopathological hallmark of tauopathies, including Alzheimer’s disease (AD), progressive supranuclear palsy (PSP), corticobasal degeneration (CBD), and Pick’s disease. While the molecular mechanisms of tau aggregation under pathological conditions are still not fully understood, biochemical evidence indicates that paired helical filaments (PHFs) extracted from the brains of AD patients co-precipitated with membrane lipids [7,8], indicating membrane-mediated tau self-assembly in vivo. Lipid membranes have been suggested to be a crucial factor in modulating amyloid nucleation and aggregation kinetics of many amyloidogenic proteins [9,10,11]. It has been reported that the interaction of tau with phospholipid membranes promotes tau misfolding and oligomerization, leading to the formation of toxic species that contribute to neurodegeneration [12]. Additionally, intracellular tau can be secreted in a vesicle-free form via direct translocation across the plasma membrane and taken up by nearby cells [13,14,15,16], further indicating a dynamic interplay between tau and neuronal membranes. Interactions between tau and lipids may not only lead to tau aggregation and propagation but also may be directly related to tau-induced cellular toxicity. Although the fundamental pathological mechanisms of tau toxicity are unclear, it has been reported that tau binding to lipid membranes leads to membrane destabilization and disruption of membrane integrity [12,17,18,19,20], similar to other amyloidogenic proteins [21,22,23]. This disruption may ultimately impair cell membrane homeostasis and trigger apoptosis. For instance, tau oligomers have been shown to effectively increase phospholipid vesicle leakage and decrease the viability of SH-SY5Y cells [24]. Dissecting the mechanistic impact of tau-membrane interactions on both membrane integrity and tau fibrillization may provide critical insights into the pathological role of tau in neurodegenerative diseases.

**Figure 1:**
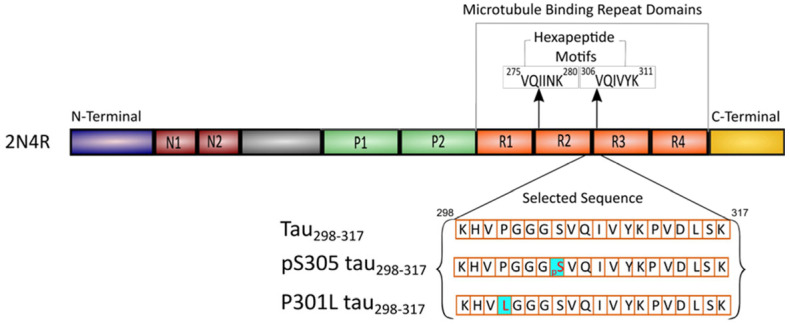
Schematic representation of the primary sequence of full-length tau (2N4R). The sequences of the fragment peptide tau_298–317_ and mutants are shown.

Short peptide fragments derived from amyloidogenic proteins have been widely employed as useful model molecules for studying the mechanisms of protein aggregation [25,26,27]. By reducing the structural complexity of full-length proteins, these model fragments enable a more focused examination of key factors driving aggregation and the associated conformational transitions. In our previous work, we used a 20-residue peptide fragment from the longest tau isoform 2N4R (residues 298–317, hereafter referred to as tau_298–317_) as a model to examine aggregation determinants within the microtubule-binding domains of tau [28]. This sequence includes the hexapeptide PHF motif (^306^VQIVYK^311^) within the microtubule-binding repeat domains of tau (Figure 1), a critical trigger for tau fibrillization through β-sheet formation [29]. The ^301^PGGG^304^ motif in the sequence has a strong tendency to form turns under normal physiological conditions [30]. The Ser305-Asp314 region also exhibits a high propensity for β-structure formation [30]. Our prior findings demonstrated that tau_298–317_ closely replicates the aggregation properties of the full-length tau [28], making it a valuable model molecule for exploring tau aggregation mechanisms. Previous studies suggest that tau fragments, generated by proteolytic cleavage, remain associated with intracellular membranes [31]. However, the influence of interactions between the crucial tau fragment peptides and lipids on membrane integrity and peptide aggregation properties remains poorly understood. In the present study, we investigate the mutual impact of the interaction between the tau fragment peptide tau_298–317_ and model phospholipid membrane vesicles with defined surface properties on both peptide-induced membrane damage and membrane-mediated peptide aggregation. In addition to the wild-type (WT) sequence, we also examined a P301L mutant (Figure 1), a genetic variant associated with frontotemporal dementia and parkinsonism linked to chromosome 17 (FTDP-17) [32]. This mutation enhances the β-sheet propensity and accelerates tau neurofibrillary tangle formation, contributing to motor and behavioral deficits in mouse models [32]. To assess the modulatory effects of phosphorylation, we also introduced a phosphate group at Ser305 in the WT peptide (Figure 1). Our study using the combination of various biophysical approaches reveals that electrostatic interactions between the peptide and lipid membrane surface play a critical role in driving tau_298–317_–membrane interactions, membrane-mediated secondary structural transition and self-assembly of the peptide, and peptide-induced membrane permeabilization.

## 2. Materials and Methods

### 2.1. Synthesis of the Peptides

Tau_298–317_ and its mutant peptides were synthesized using a PS3 solid-phase peptide synthesizer (Protein Technologies Inc., Woburn, MA, USA) with Fmoc chemistry and Rink amide AM resin [33]. The crude peptides were purified using high-performance liquid chromatography (HPLC) on a C18 reverse-phase column. The molecular weight of the peptides was confirmed by matrix-assisted laser desorption ionization (MALDI) mass spectrometry. The purified peptides were lyophilized and stored at −80 °C. Before use, the peptide aliquots were dissolved in Milli-Q water, and the peptide concentration was determined using the Tyr UV absorbance at 280 nm (ε = 1280 M^−1^ cm^−1^).

### 2.2. Lipid Vesicle Preparation

Small unilamellar vesicles (SUVs) of POPC (1-palmitoyl-2-oleoyl-sn-glycero-3-phosphocholine) and POPG (1-Palmitoyl-2-oleoyl Phosphatidylglycerol) were prepared using a thin film-membrane extrusion method described previously [34]. Briefly, appropriate volumes of phospholipid solution dissolved in chloroform (5 mg/mL) were transferred to a round-bottom flask, and a thin lipid film was formed by solvent evaporation under vacuum. The film was then hydrated with 50 mM Na-phosphate buffer (pH 7.4), followed by three freeze-thaw cycles (20 min each) to reduce vesicle lamellarity. The final SUVs were obtained by 20 times extrusion through the polycarbonate membrane (50 nm pore size). To prepare carboxyfluorescein (20 mM) encapsulated SUVs, the lipid film was hydrated by adding pH 7.4 buffer containing 50 mM carboxyfluorescein, followed by the same thin-film membrane extrusion procedure. Free carboxyfluorescein was removed by passing the vesicle and carboxyfluorescein mixture solution through a Sephadex G-50 size-exclusion column pre-equilibrated with pH 7.4 phosphate buffer. Phospholipid concentrations were determined using the Bartlett assay for POPG and the Stewart assay for POPC [35,36].

### 2.3. Membrane Leakage Study

One hundred twenty μL of carboxyfluorescein-encapsulating lipid vesicles containing 100 μM lipid was loaded into a disposable fluorescence cuvette. Baseline fluorescence was recorded for 3 min at 20 s intervals with an excitation wavelength of 493 nm and an emission wavelength of 520 nm using a Horiba FluoroMax-4 spectrofluorometer. After that, an appropriate amount of peptide stock solution was added, and fluorescence intensity was monitored for 15 min. Then, 1% (*v*/*v*) Triton X-100 was added to fully lyse the vesicles, releasing all encapsulated dye to achieve maximum fluorescence intensity. Each experiment was repeated three times. The membrane leakage was evaluated based on the leakage of the dye from the liposomes using the equation below:

$$Fraction\;of\;dye\;released=\frac{\left(I_{t}-{\;I}_{0}\right)}{\left(I_{100}-I_{0}\right)},$$

where $I_{t}$ is the fluorescence intensity of the dye-containing SUVs, $I_{0}$ is the baseline fluorescence signal intensity in the absence of peptides, and $I_{100}$ is the maximum fluorescence intensity obtained after adding Triton X-100.

### 2.4. Particle Size Measurement Using Dynamic Light Scattering (DLS)

Hydrodynamic radius measurements of the lipid vesicles were conducted using a Zetasizer Nano ZS instrument (Malvern Instruments, Malvern, UK) equipped with a 633 nm laser [33]. A 100 µL vesicle sample solution was added to a disposable polystyrene cuvette, and the measurement was carried out at 25 °C, with triplicate repeats consecutively computed from 12–15 runs for each measurement. For kinetic studies, the hydrodynamic diameter was monitored for 1 h, and the Z-average value was obtained from 12 runs with 3-min intervals.

### 2.5. Circular Dichroism (CD) Study of the Peptides

Peptide samples (50 µM) were prepared in pH 7.4 phosphate buffer (10 mM) with or without 100 µM phospholipid vesicles [33]. CD measurements were taken at different incubation times. During the measurement, a 300 μL aliquot was loaded into a 0.1 cm quartz cell, and CD spectra were recorded on a JASCO J-810 spectropolarimeter at a bandwidth of 1.0 nm, a scanning speed of 100 nm/min, a resolution of 0.1 nm, and an averaging time of 2 s. A buffer control was run as a blank.

### 2.6. Aggregation Kinetics Study of the Peptides

The aggregation kinetics of the peptides were conducted with or without 100 µM POPG or POPC SUVs in pH 7.4 buffer (50 mM Na-phosphate) containing 20 µM ThT [33]. Samples were transferred to a 96-well plate (100 µL/well), sealed with a lid and parafilm, and loaded into a Gemini SpectraMax EM fluorescence plate reader (Molecular Devices, Sunnyvale, CA, USA) for kinetic analysis. The excitation and emission wavelengths were set to 440 nm and 480 nm, respectively, with the temperature maintained at 25 °C. ThT fluorescence intensity was recorded every 10 min, with a 5-s shaking step before each measurement. Each experiment was performed in triplicate.

### 2.7. Atomic Force Microscopy (AFM) Measurement

Peptide aliquots from kinetics assays with or without 100 μM phospholipid vesicles were deposited onto freshly cleaved mica (8 × 8 mm) and incubated in a sealed box in the dark overnight to dry. AFM images were acquired using an Asylum Research MFP-3D Bio AFM system with Mikromasch NSC15/AI BS cantilevers. Tapping-mode scans were conducted at a tip scan rate of 1.0 Hz with a cantilever drive frequency of 70 kHz. Image processing was performed using Gwyddion software (version 2.61).

## 3. Results and Discussion

### 3.1. Characterization of the Aggregation Properties of Tau_298–317_ Mutants

**Figure 2:**
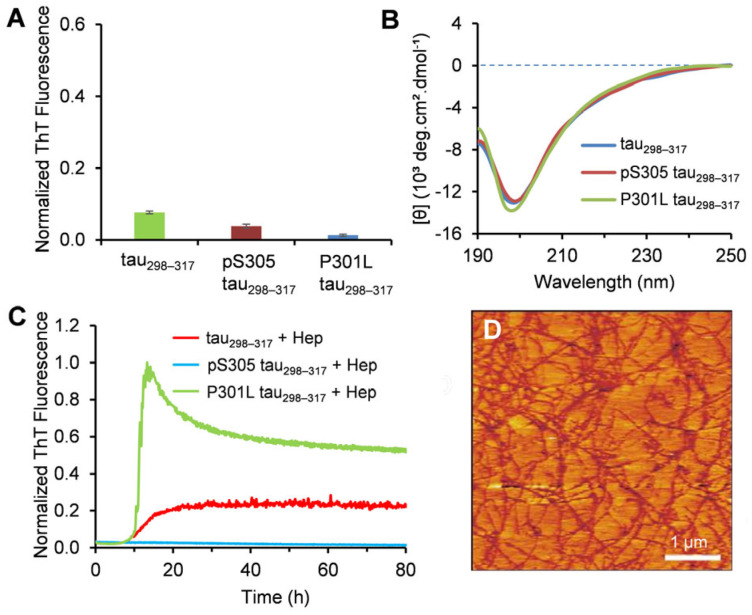
(**A**) Normalized ThT fluorescence intensity at 48 h of the aggregation kinetics of tau_298–317_ and the mutants (12.5 µM) measured at 25 °C in pH 7.4 buffer (50 mM Na-phosphate). Data are reported as mean ± standard deviation of triplicate results. (**B**) CD spectra of tau_298–317_ and the mutants (50 μM) recorded after 48 h of incubation at 25 °C in pH 7.4 buffer (10 mM Na-phosphate). (**C**) Aggregation kinetics of tau_298–317_ and the mutants (12.5 μM) in the presence of 1.5 μM Hep monitored by ThT fluorescence at 25 °C in pH 7.4 buffer (50 mM Na-phosphate). (**D**) Tapping-mode AFM images of the P301L tau_298–317_ sample taken at the endpoint of the aggregation kinetics experiment.

Isolated tau fragment molecules from the crucial microtubule-binding domain have been reported as valuable models for elucidating the molecular mechanism of tau aggregation [37,38]. Prior characterization studies of tau_298–317_ revealed that the peptide adopts a random coil conformation and does not spontaneously aggregate into amyloid fibrils in an aqueous solution without additional aggregation-promoting cofactors [28], similar to full-length tau. To assess the aggregation propensity of the phosphorylated tau_298–317_ and the P301L mutant, we first examined the fibrillization kinetics using the ThT fluorescence assay. ThT fluoresces at 480 nm upon binding to amyloid fibrils and is widely used for probing protein amyloid formation [39]. As shown in Figure 2A, negligible ThT fluorescence was observed for both the WT tau_298–317_ and the mutants after 48 h, indicating no fibril formation during the study period. AFM imaging of the peptide samples collected from the aggregation kinetics experiments showed no oligomers or fibrils formed for tau_298–317_ and the mutants (Figure S1), consistent with the ThT fluorescence results. The secondary structure properties of the peptides were further assessed using CD spectroscopy. The CD spectra of the WT tau_298–317_ and the mutants showed a minimum below 200 nm after 48 h incubation (Figure 2B), suggesting that the peptides predominantly retain a random coil conformation and do not transition into ordered β-sheet-rich structures. This is consistent with the properties of the full-length tau, which is hydrophilic, highly soluble in aqueous environments, and does not spontaneously aggregate under normal physiological conditions [40].

The in vitro aggregation of tau is usually induced by external factors such as glycosaminoglycans (GAGs), ribonucleic acids, and fatty acids [41,42,43], which may act as catalytic scaffolds to reduce the energy barrier of nucleation-dependent amyloidogenesis. Prior work demonstrated that heparin (Hep), a negatively charged and sulfated polysaccharide, promotes the aggregation of tau_298–317_ while failing to induce fibril formation of pS305 tau_298–317_ [28]. The results in the present study showed that Hep also significantly enhances the aggregation of P301L tau_298–317_ (Figure 2C). A fast growth phase in aggregation kinetics was observed when P301L tau_298–317_ (12.5 μM) was co-incubated with Hep (1.5 μM). The maximum fluorescence intensity was approximately 4.4 times that of the WT tau_298–317_. AFM imaging further confirmed the formation of dense amyloid fibrils in the P301L tau_298–317_ samples (Figure 2D). These results indicate that the P301L mutation significantly increases the aggregation propensity of the fragment peptide, which is consistent with previous reports that the mutation also promotes aggregation of full-length tau [44,45].

### 3.2. Effect of Tau_298–317_ and the Mutants on Leakage of Zwitterionic POPC Vesicles

**Figure 3:**
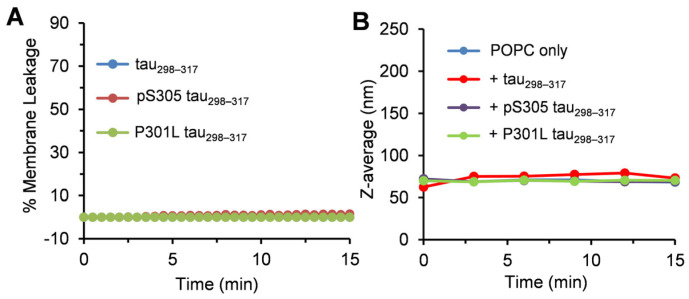
(**A**) Membrane leakage of POPC vesicles (100 µM) induced by tau_298–317_, pS305 tau_298–317_, or P301L tau_298–317_ (12.5 µM) at 25 °C in pH 7.4 buffer (50 mM Na-phosphate). (**B**) Hydrodynamic size of POPC vesicles (100 µM) measured by DLS at different time points in the presence of 12.5 µM tau_298–317_, pS305 tau_298–317_, or P301L tau_298–317_.

Maintaining cell membrane integrity is essential for normal ion homeostasis and signal transduction across the cell membrane. Neuronal membrane damage due to interactions with various amyloidogenic proteins, such as Aβ, α-synuclein, and tau, has been implicated in neuronal toxicity [46,47,48]. Therefore, a mechanistic understanding of the interactions between tau and phospholipids may provide essential insights into the pathophysiology of tauopathies. Here, we first used liposomes composed of zwitterionic POPC as model membranes and assessed the peptide-induced membrane disruption using a dye leakage assay. Phosphatidylcholine (PC) is the most abundant phospholipid in eukaryotic cell membranes [49]. As shown in Figure 3A, adding 12.5 μM tau_298–317_ peptide or the mutants did not induce observable dye release, indicating that the peptides do not strongly interact with zwitterionic POPC to impair membrane integrity. To further validate these findings, we used dynamic light scattering (DLS) to monitor the change in liposome hydrodynamic diameter (Z-avg) over time. Consistent with the dye leakage results, no significant change in vesicle size was observed (Figure 3B and Fiigure S2), suggesting minimal interaction between tau_298–317_ (or its mutants) and POPC vesicles.

Previous studies on Aβ40 and dipalmitoylphosphatidylcholine (DPPC) demonstrated that electrostatic interactions between the negatively charged Glu11 residue of Aβ40 and the tertiary amine head groups of DPPC promoted peptide adsorption onto vesicle surfaces [34,50]. In contrast, tau_298–317_ has a net positive charge of 3.1 at neutral pH, which likely induces electrostatic repulsion with the POPC head group, thus preventing peptide and membrane interactions. This is consistent with previous reports that the full-length tau (2N4R), which also has a net positive charge, fails to bind favorably to zwitterionic dimyristoylphosphatidylcholine (DMPC) membranes [51].

### 3.3. Effect of Tau_298–317_ and the Mutants on Leakage of Anionic POPG Vesicles

**Figure 4:**
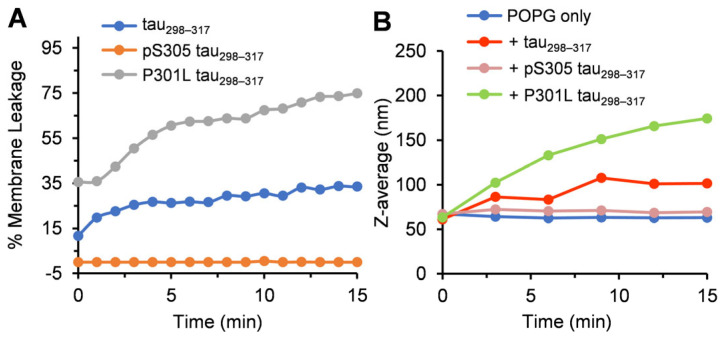
(**A**) Membrane leakage of POPG vesicles (100 µM) induced by tau_298–317_, pS305 tau_298–317_, or P301L tau_298–317_ (12.5 µM) at 25 °C in pH 7.4 buffer (50 mM Na-phosphate). (**B**) Hydrodynamic size of POPG vesicles (100 µM) measured by DLS at different time points in the presence of 12.5 µM tau_298–317_, pS305 tau_298–317_, or P301L tau_298–317_.

We next investigated the effect of the tau peptides on the leakage of lipid vesicles containing POPG with an anionic headgroup. Alzheimer’s patients showed higher levels of anionic phospholipids, and the microtubule-binding repeats of tau have been reported to interact with anionic lipid membranes [19,52]. Our results showed that tau_298–317_ (12.5 µM) rapidly induced POPG membrane leakage, with ~34% dye release observed in 15 min (Figure 4A). Upon addition of tau_298–317_, the hydrodynamic diameter of POPG vesicles increased by ~1.6-fold, from 63 nm to 102 nm in 15 min (Figure 4B), suggesting rapid adsorption of tau_298–317_ onto the vesicle surface and the formation of tau_298–317_–POPG complexes. The vesicle size enlargement slowed down over time, reaching ~116 nm after 1 h, which may be attributed to the reduction of free peptides in solution after adsorption onto the vesicle surface. In contrast, pS305 tau_298–317_ did not induce leakage of the POPG vesicles (Figure 4A), and the hydrodynamic diameter of the vesicles remained unchanged (Figure 4B), indicating a lack of interaction between the mutant peptide and the lipid vesicles. The negatively charged phosphate group at Ser305 likely introduces electrostatic repulsion with the anionic headgroup of POPG, thus preventing peptide-membrane interaction. Notably, phosphorylation at Ser305 is adjacent to the hydrophobic PHF6 hexapeptide motif (^306^VQIVYK^311^) (Figure 1), which plays a crucial role in the nucleation of tau filament formation and strongly favors β-sheet formation [29,53,54]. Phosphorylation close to this site may also disrupt the intermolecular hydrophobic interactions of the hexapeptide motif, contributing to the inhibition of both heparin-induced peptide aggregation and the membrane-disrupting activity observed in this study.

The different effects of tau_298–317_ on the leakage of POPG and POPC liposomes suggest the importance of electrostatic interactions between the peptide and membrane surface in membrane–peptide interactions [55,56]. Previous studies on lipid monolayer disruption by full-length tau (2N4R) support this finding, showing that while tau does not associate with zwitterionic DMPC lipids, it exhibits strong electrostatic coupling with the anionic DMPG monolayer [51]. Similarly, the anionic head group of POPG was shown to participate in electrostatic interactions with positively charged Lys residues in the non-amyloid-β component (NAC) region of α-synuclein [57,58]. Our previous study also showed that tau_298–317_ interacts with anionic heparin through Lys-mediated electrostatic interactions [28]. The electrostatic interaction between the Lys residues in the peptide and the anionic POPG head group likely localizes the peptide near the membrane surface. Adsorption of the peptide onto the POPG surface would further neutralize charges on both components, increasing the overall hydrophobicity of the vesicle surface. This charge neutralization may lead to thermodynamic instability in the hydrophilic environment, causing vesicle aggregation or collapse.

The dye leakage study further demonstrated that the P301L mutant caused significantly stronger membrane disruption, with POPG vesicle dye release more than doubled in 15 min compared to tau_298–317_ (75% vs. 34%) (Figure 4A). The hydrodynamic diameter of POPG vesicles increased significantly upon the addition of 12.5 µM P301L tau_298–317_, from 63 nm to 174 nm at 15 min (Figure 4B), larger than the size increase observed with tau_298–317_. This suggests more extensive peptide-induced liposome aggregation or lipid reaggregation following membrane disruption.

### 3.4. Effect of Lipid Membrane on the Aggregation of Tau Fragment

**Figure 5:**
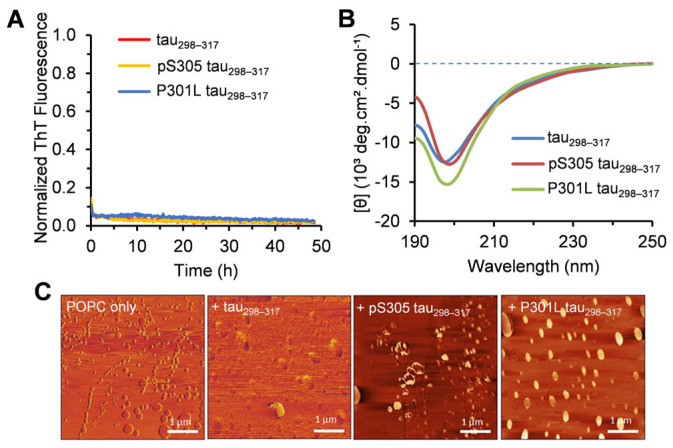
(**A**) Aggregation kinetics of tau_298–317_ and the mutants (12.5 µM) in the presence of 100 µM POPC monitored by ThT fluorescence at 25 °C in pH 7.4 buffer (50 mM Na-phosphate). (**B**) CD spectra of 50 μM tau_298–317_ and the mutants after 48 h of co-incubation with 100 µM POPC at 25 °C in pH 7.4 buffer (10 mM Na-phosphate). (**C**) Tapping-mode AFM images of POPC alone and POPC–peptide mixtures collected at the endpoint of the aggregation kinetics experiment shown in (**A**).

The peptide–membrane interactions have a dual effect on both membrane integrity and peptide self-association. The short hexapeptide motifs in tau, including ^306^VQIVYK^311^ in the third repeat, can form the core of PHF by stacking cross-β-sheets [29,53]. The plasma membrane has been considered as a potential initiating place for PHF assembly within the cell [59]. To investigate the effects of lipid vesicles on the aggregation of tau_298–317_ and the mutants, we monitored their fibrillization kinetics in the presence of additional lipid vesicles. As shown in Figure 5A, with POPC, ThT fluorescence remained minimal throughout the measurement period, indicating no amyloid fibril formation. After 48 h incubation with POPC, the CD spectra of tau_298–317_ and the mutants showed a minimum at ~197 nm (Figure 5B), indicating a predominantly random coil structure. AFM imaging further showed that only POPC liposomes were observed without detectable fibrillar structures (Figure 5C). These results suggest that POPC vesicles have a minimal impact on tau fragment aggregation, thereby further validating the weak interaction between peptides and POPC membranes revealed in the membrane leakage study.

**Figure 6:**
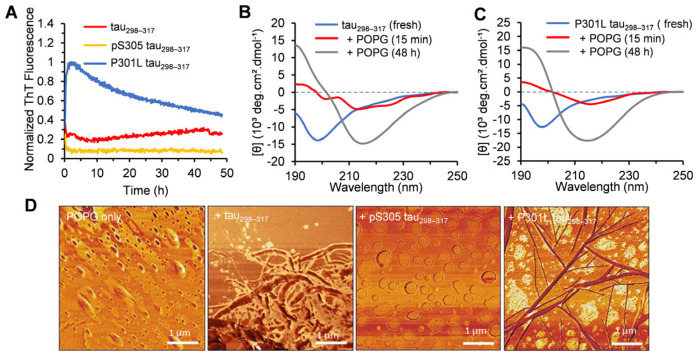
(**A**) Aggregation kinetics of tau_298–317_ and the mutants (12.5 µM) in the presence of 100 µM POPG monitored by ThT fluorescence at 25 °C in pH 7.4 buffer (50 mM Na-phosphate). (**B**) CD spectra of fresh tau_298–317_ (50 μM) and after co-incubation with 100 µM POPG at 25 °C in pH 7.4 buffer (10 mM Na-phosphate). (**C**) CD spectra of fresh P301L tau_298–317_ (50 μM) and after co-incubation with 100 µM POPG at 25 °C in pH 7.4 buffer (10 mM Na-phosphate). (**D**) AFM images of POPG alone and POPG–peptide mixtures collected at the endpoint of the aggregation kinetics experiment shown in (**A**).

The aggregation kinetics of tau_298–317_ in the presence of POPG were also monitored using ThT fluorescence. The results showed a gradual increase in the fluorescence for the peptide (12.5 μM) co-incubated with POPG (Figure 6A), although the overall fluorescence intensity remained low, indicating the formation of fibrillar aggregates. AFM imaging also showed the formation of short fibrils of tau_298–317_ at the endpoint of the aggregation kinetics (Figure 6D). These results suggest that, unlike POPC, POPG vesicles promote the self-association of the peptide to form amyloid fibrils. The CD spectra showed that after incubation with POPG for 15 min, the intensity of the negative band at ~198 nm, observed in the fresh sample with predominantly random coil structures, decreased significantly (Figure 6B), indicating a reduction in unordered conformations. Additionally, two negative bands at ~213 and 223 nm were observed, which could be attributed to the formation of transient helical and/or turn structures upon peptide adsorption to the liposomes [60]. Ait-Bouziad et al. also reported that interactions between tau and phosphatidylserine vesicles induce transient α-helical structures in one or more repeat regions of tau (R1–R4), promoting tau-phospholipid complex formation [12]. After 48 h incubation, the CD spectrum displayed a pronounced negative peak at 216 nm and a positive band at 192 nm (Figure 6B), suggesting the formation of β-sheet-rich amyloid structures.

The aggregation of P301L tau_298–317_ (12.5 µM) was significantly accelerated by the addition of POPG (100 µM) (Figure 6A). The ThT fluorescence intensity increased immediately in the aggregation kinetics study and reached the maximum within 2 h, with a final fluorescence intensity significantly higher than that of the WT peptide (Figure 6A). Amyloid fibril structures were also observed in the AFM imaging (Figure 6D), consistent with the ThT results. The CD spectrum of the peptide showed a broad negative band with a minimum at 216 nm after 15 min of mixing with POPG (Figure 6C), indicating a fast conformational transition from random coils to β-sheet structures after binding to the membrane, without the formation of α-helical intermediate structures. After 48 h incubation, the CD spectrum exhibited a strong negative band at 215 nm and a positive band at 192 nm (Figure 6C), further confirming the dominant β-sheet structures in mature amyloid fibrils. In the aggregation kinetics of the peptides at higher concentrations (e.g., 50 µM and 100 µM), the ThT intensity of both the WT and P301L mutant peptides increased rapidly, consistent with the formation of densely packed fibrillar structures observed in AFM imaging (Figure S3). The DLS results further showed that increasing the concentration of tau_298–317_ or the P301L mutant significantly increased the vesicle size (Figure S4). The addition of 50 μM tau_298–317_ peptide to POPG increased vesicle diameter from 63 nm to 534 nm within 15 min. After 60 min, the mixture of POPG and tau_298–317_/P301L mutant peptide (50 µM or 100 µM) formed large clumps with hydrodynamic diameters greater than 2 μm (Figure S4). These large particles may represent aggregated peptides, reaggregation of free lipids after the rupture of POPG vesicles, or aggregates composed of mixtures of peptides and lipids. In contrast, pS305 tau_298–317_ (up to 100 μM) did not significantly alter the liposome size (Figure S4), consistent with its minimal interaction with the lipid vesicles. No increase in ThT fluorescence (Figure 6A and Figure S3) or amyloid fibrils in AFM imaging (Figure 6D and Figure S3) was observed for pS305 tau_298–317_. CD spectroscopy also showed a predominantly random coil conformation after 48 h of incubation with POPG (Figure S5). These results suggest that, similar to POPC, POPG vesicles do not promote the aggregation of the pS305 tau_298–317_ either.

### 3.5. Crucial Interactions in Mediating Membrane Leakage and Peptide Aggregation

Our results suggest a pronounced dual impact of interactions between tau_298–317_ and lipid vesicles, in particular electrostatic interactions, on both membrane leakage and peptide aggregation. Tau_298–317_ contains 3 Lys and 1 His residues and has a net positive charge of 3.1 at neutral pH. The positively charged groups in tau_298–317_ play a crucial role in electrostatic interactions with heparin to initiate aggregation [28]. PC lipids have a zwitterionic head group composed of a positively charged quaternary amine and a negatively charged phosphate group. Prior studies indicate that in DPPC vesicles, the amine group is more solvent-exposed, while the phosphate group is more shielded in the inner layer of the membrane surface, making the amine more accessible for early interactions with Aβ peptides [34,50]. Thus, the unfavorable repulsive electrostatic interactions of the exposed amine group at the POPC vesicle surface likely prevent the close proximity and adsorption of tau_298–317_ on the membrane, consistent with the absence of peptide-induced membrane damage or membrane-mediated aggregation. While phosphorylation in the pS305 mutant reduces the overall positive charge of the peptide, it does not improve peptide interaction with POPC significantly, as neither lipid leakage nor peptide aggregation was observed.

**Figure 7:**
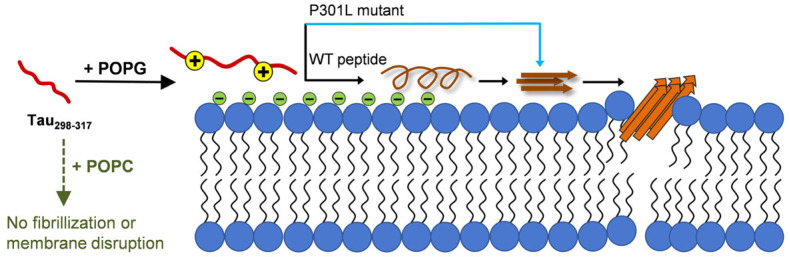
Schematic representation of a proposed process of membrane–tau_298–317_ interaction-induced peptide aggregation and membrane disruption.

In contrast, POPG has a negatively charged phosphate head group, which strongly interacts with Lys and His residues in tau_298–317_ through electrostatic attractions. It has been reported that substituting Lys311 with Ala (K311A) in the PHF6 hexapeptide region of tau significantly weakens the complex formation of tau and negatively charged phosphatidylserine vesicles [12]. The rapid dye leakage and increase in hydrodynamic diameter upon adding tau_298–317_ to POPG vesicles suggest strong interactions, especially electrostatic interactions that drive peptide–membrane complex formation. As illustrated in a proposed schematic model (Figure 7), peptide adsorption onto the membrane surface may induce a conformation conversion from a disordered state to more ordered structures containing a transient α-helical conformation. The localized accumulation of tau_298–317_ with an ordered structure on the membrane surface reduces intermolecular electrostatic repulsion and nucleation barriers, facilitating subsequent self-association into oligomers and larger β-sheet-rich fibrillar structures, which further lead to membrane leakage. Although our current data could not directly identify the specific tau fragment aggregate species responsible for membrane disruption, the rapid onset of vesicle leakage suggests that early-formed, small oligomeric species are the most likely mediators of membrane damage. Phosphorylation on the peptide inhibits both peptide-induced membrane damage and membrane-mediated peptide aggregation, further suggesting the critical role of electrostatic interactions in early peptide-membrane binding and the subsequent effects on membrane integrity and peptide aggregation.

The PGGG motif in the microtubule-binding repeat domains of tau has been suggested to have a helical propensity or form type II β-turns [30], in agreement with our CD results showing a rapid conformational transition of tau_298–317_ to form ordered helical or turn structures after mixing with POPG lipids. The Pro residue in this motif introduces a kink in the amide bond, restricting the extension of the high propensity of β-structures of the adjacent PHF hexapeptide region. This structural constraint may create a U-shaped feature that favors an intramolecular antiparallel β-sheet conformation, thus increasing the energetic barrier for intermolecular interactions of the crucial PHF region at the membrane interface, thereby limiting peptide self-association to form oligomers and fibrils. The P301L mutation eliminates this conformational constraint, facilitating the conversion of the membrane-bound peptide into ordered β-sheet-rich structures without the formation of an α-helical intermediate conformation (Figure 7). This transition further favors the assembly of the high aggregation-prone ^306^VQIVYK^311^ hexapeptide region to form oligomers and larger fibrils, which likely contributes to enhanced membrane damage activity compared to the WT peptide. Previous simulation studies reported that Pro or Gly kinks in helical amphiphilic peptides also hinder barrel-stave pore formation by disrupting the compact packing of peptides within barrel-stave pores [61]. Additionally, the more positive GRAVY (grand average of hydropathy) score of P301L tau_298–317_ compared to tau_298–317_ (0.065 vs. −0.205) suggests that the enhanced interaction of P301L tau_298–317_ with POPG may involve not only electrostatic interactions but also hydrophobic interactions with the acyl chains of POPG. The P301L mutation specifically increases local hydrophobicity near the PHF region, which may further promote membrane-mediated peptide self-assembly and the insertion of aggregated species into the hydrophobic tails of lipids, resulting in significantly enhanced membrane damage. A previous study also showed that the N-terminal cationic PHF6 (NH3^+^-PHF6) interacts electrostatically with DMPG and produces slower membrane disruption, whereas the neutralized PHF6 (acetylated PHF6, Ac-PHF6) shows a stronger membrane-disrupting ability by inserting into the interior of the DMPG membrane [62]. Our results suggest the important roles of both electrostatic and hydrophobic interactions in directing the formation of the membrane–peptide complex and subsequent peptide aggregation and membrane damage.

## 4. Conclusions

In this study, we investigated the dual effects of the membrane interaction of a tau fragment, tau_298–317_, derived from the tau critical microtubule-binding domain, on peptide-induced membrane disruption and membrane-mediated peptide self-assembly. Our results show that WT tau_298–317_ and the two mutants (P301L and pS305) do not interact with zwitterionic POPC membranes, likely due to electrostatic repulsion with the positively charged amine residue in the POPC headgroup, resulting in neither membrane leakage nor peptide aggregation. In contrast, tau_298–317_ favorably interacts with negatively charged POPG liposomes, and peptide adsorption on the membrane surface leads to localized peptide condensation and a rapid conformational shift of the peptide from random coil to α-helical intermediate, which further promotes peptide self-association to form β-sheet-rich fibrillar structures. The rapid POPG membrane leakage indicates a synergistic process of membrane-mediated peptide aggregation and peptide-induced membrane disruption. Phosphorylation at Ser305 interferes with favorable electrostatic interactions with POPG, thereby preventing both peptide aggregation and membrane disruption. In contrast, the P301L mutation significantly enhances both membrane-mediated aggregation and peptide-induced POPG membrane leakage, likely due to the mutation-induced release of local conformational constraints, which facilitates fast conformational conversion to β-sheet structures. This study provides mechanistic insights into the molecular mechanisms of the membrane-mediated aggregation of crucial local regions of tau and the aggregation-induced membrane disruption, which may be critical for the development of potential strategies to mitigate tau aggregation and its associated neurotoxicity. Given that the early contact between tau_298–317_ and POPG is primarily driven by electrostatic interactions, targeting these interactions may represent a promising approach to prevent both membrane-mediated tau aggregation and aggregation-induced membrane rupture.

## Data Availability

The datasets used during the current study may be made available by the corresponding author upon reasonable request.

## Supplementary Materials

The following supporting information can be downloaded at: https://www.mdpi.com/article/10.3390/membranes15070208/s1. Figure S1: Tapping-mode AFM images of tau_298–317_ (A), pS305 tau_298–317_ (B), and P301L tau_298–317_ (C) samples collected at the end of the aggregation kinetics experiment. Figure S2: Time-dependent changes in the hydrodynamic size of POPC (100 µM) vesicles in the absence or the presence of different concentrations of tau_298–317_ (A), pS305 tau_298–317_ (B), or P301L tau_298–317_ (C) in pH 7.4 buffer (50 mM Na-phosphate). Figure S3: (A) Normalized aggregation kinetics of 50 µM tau_298–317_ and the mutants in the presence of 100 µM POPG followed by ThT fluorescence at 25 °C in pH 7.4 buffer (50 mM Na-phosphate). (B) Normalized aggregation kinetics of 100 µM tau_298–317_ and the mutants in the presence of 100 µM POPG. (C) AFM images of 100 µM tau_298–317_ and the mutants in the presence of POPG. The samples for AFM were collected at the end of the aggregation kinetics shown in (B). Figure S4: Time-dependent changes in the hydrodynamic size of POPG (100 µM) vesicles in the absence or the presence of different concentrations of tau_298–317_ (A), pS305 tau_298–317_ (B), or P301L tau_298–317_ (C) in pH 7.4 buffer (50 mM Na-phosphate). Figure S5: CD spectra of 50 μM pS305 tau_298–317_ in the presence of 100 μM POPG obtained after 48 h of incubation at 25 °C in pH 7.4 buffer (10 mM Na-phosphate).
