# Supplementary material for: Effect of Tau Fragment and Membrane Interactions on Membrane Permeabilization and Peptide Aggregation
Source: Membranes (Basel). 2025 Jul 13;15(7):208. doi: 10.3390/membranes15070208 (PMC12297872; doi:10.3390/membranes15070208)
Supplement: Supplementary file 1 [file membranes-15-00208-s001.zip › membranes-3715801-supplementary.pdf]

Supporting Information

Effect of Tau Fragment and Membrane Interactions on Membrane Permeabilization and Peptide Aggregation

Majedul Islam [1], Md Raza Ul Karim [1], Emily Argueta [1], Mohammed N. Selim [1], Ewa P. Wojcikiewicz [2] and Deguo Du [1,*]

1 Department of Chemistry and Biochemistry, Florida Atlantic University, Boca Raton, FL 33431, USA

2 Department of Biomedical Science, Charles E. Schmidt College of Medicine, Florida Atlantic University, Boca Raton, FL 33431, USA

* Correspondence: ddu@fau.edu

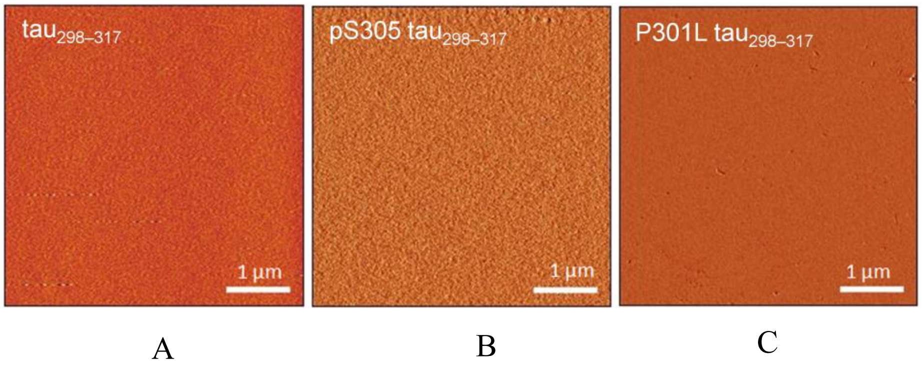

Figure S1. Tapping-mode AFM images of tau$_{298–317}$ (A), pS305 tau$_{298–317}$ (B), and P301L tau$_{298–317}$ (C) samples collected at the end of the aggregation kinetics experiment.

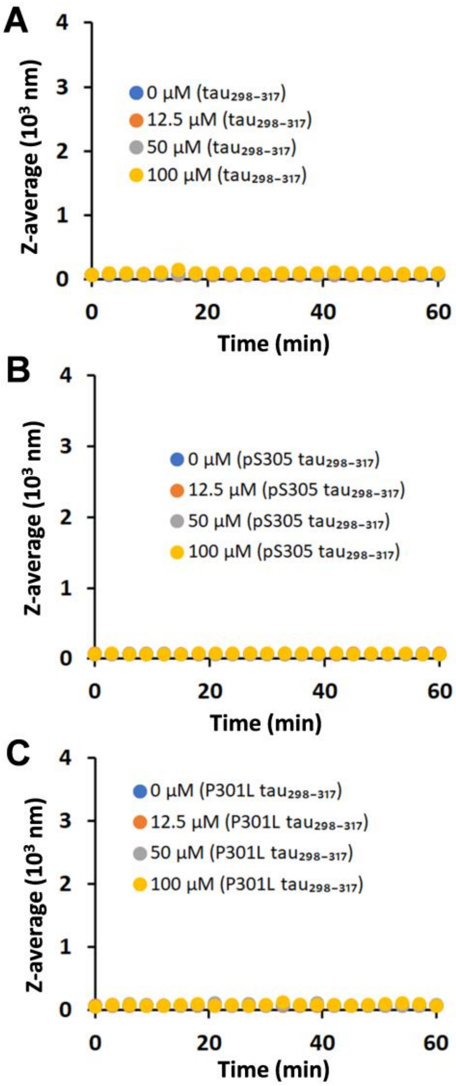

Figure S2. Time-dependent changes in the hydrodynamic size of POPC (100 µM) vesicles in the absence or the presence of different concentrations of $tau_{298–317}$ (A), pS305 $tau_{298–317}$ (B), or P301L $tau_{298–317}$ (C) in pH 7.4 buffer (50 mM Na-phosphate).

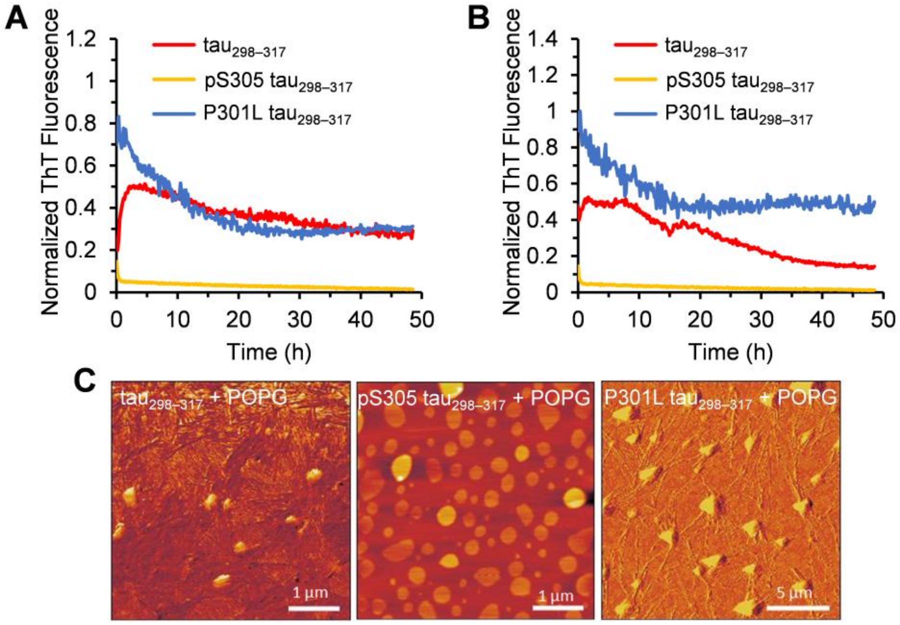

Figure S3. (A) Normalized aggregation kinetics of 50 µM tau$_{298–317}$ and the mutants in the presence of 100 µM POPG followed by ThT fluorescence at 25 °C in pH 7.4 buffer (50 mM Na-phosphate). (B) Normalized aggregation kinetics of 100 µM tau$_{298–317}$ and the mutants in the presence of 100 µM POPG. (C) AFM images of 100 µM tau$_{298–317}$ and the mutants in the presence of POPG. The samples for AFM were collected at the end of the aggregation kinetics shown in (B).

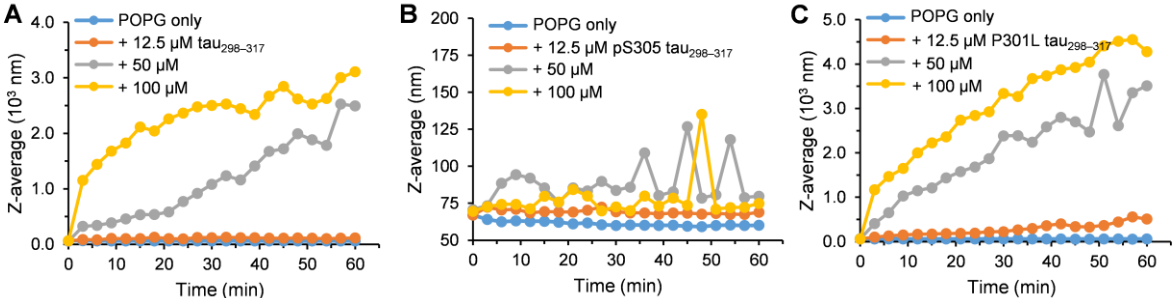

Figure S4. Time-dependent changes in the hydrodynamic size of POPG (100 µM) vesicles in the absence or the presence of different concentrations of tau$_{298–317}$ (A), pS305 tau$_{298–317}$ (B), or P301L tau$_{298–317}$ (C) in pH 7.4 buffer (50 mM Na-phosphate).

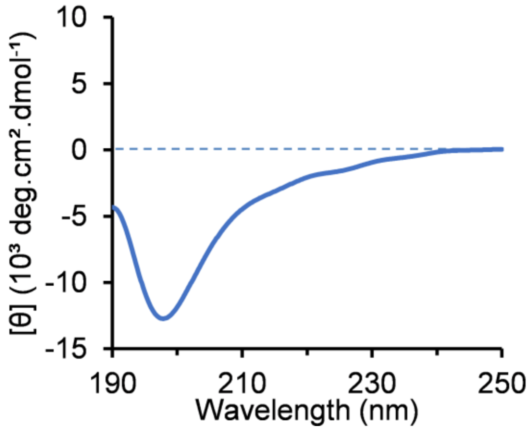

Figure S5. CD spectra of 50 μM pS305 tau$_{298–317}$ in the presence of 100 μM POPG obtained after 48 h of incubation at 25 °C in pH 7.4 buffer (10 mM Na-phosphate).
